# Integrative Approaches in Computational Biomedical Imaging

**DOI:** 10.1155/2012/162892

**Published:** 2012-12-30

**Authors:** Huafeng Liu, Pengcheng Shi, Yunmei Chen

**Affiliations:** ^1^State Key Laboratory of Modern Optical Instrumentation, Zhejiang University, Hangzhou 310027, China; ^2^B. Thomas Golisano College of Computing and Information Sciences, Rochester Institute of Technology, Rochester, NY 14623, USA; ^3^Department of Mathematics, University of Florida, 458 Little Hall, Gainesville, FL 32611-8105, USA

Increasingly wider availability of biomedical imaging modalities, such as X-ray computed tomography (CT), magnetic resonance imaging (MRI), ultrasonic imaging, positron emission tomography (PET), single-photon emission computed tomography (SPECT), and optical imaging, have led to significant progresses in both biomedical research and clinical practice. Advancements in imaging technology and image informatics have facilitated and enabled quantitative understanding of the biological structures, functional organisms, and pathological mechanisms.

This special issue presents 14 latest research contributions in computational biomedical imaging. The focuses have been on the computational principles, strategies, methods, and algorithms that create, extract, and understand imaging data of biomedical significance. 

The first group of papers has showcased the development and applications of rigorous optimization techniques in biomedical imaging. The work based on dual unscented Kalman filtering provides a reliable and efficient platform to understand the BOLD signals from fMRI. In the other paper, the robust discrete particle swarm optimization algorithm is adopted to solve the branch-cut phase unwrapping problem of MRI data. Without the need for a regularization penalty term, a semigreed method for bioluminescence tomography reconstruction is fast and stable for small animal imaging.

The second group of papers has addressed image segmentation and tissue characterization problems, ever popular among image analysis researchers. The work of L. Li et al. applies machine learning techniques to characterize lymph node metastasis in gastric cancer from gemstone spectral imaging with much improved outcomes, while G. V. Sanchez. Ferrero et al. make new advances in ultrasonic speckle characterization using generalized Gama distribution and generalized Gama mixture model. 

In recent years, physically meaningful modeling and simulation have become integral components for model-constrained imaging and model-based image understanding. Thus, the third group of papers has continued the stride in this direction. The propagation of myocardial electrical activation is simulated using a monodomain model, without the need of explicit mesh constraints. In similar spirit, one work performs forward modeling of 3D optical molecular imaging with simplified spherical harmonics approximation using extended finite element method. 

The final group of papers has highlighted some new imaging modalities and their potential use in clinical practice. The further development of ultrasound-based quantitative imaging of Young's modulus with high accuracy is presented in this issue. And the novel Diffusion Kurtosis Imaging shows that more detailed classification of brain tissues is clinical significance.

We appreciate the many high quality submissions to this special issue from leading researchers in computational biomedical imaging. Because of space limitation, we can only accept a small number of most exciting works. We sincerely hope that the remaining interesting research outcomes will be soon published in other venues.

